# Opportunities for natural infrastructure to improve urban water security in Latin America

**DOI:** 10.1371/journal.pone.0209470

**Published:** 2018-12-21

**Authors:** Beth Tellman, Robert I. McDonald, Joshua H. Goldstein, Adrian L. Vogl, Martina Flörke, Daniel Shemie, Russ Dudley, Rachel Dryden, Paulo Petry, Nathan Karres, Kari Vigerstol, Bernhard Lehner, Fernando Veiga

**Affiliations:** 1 School of Geographical Sciences and Urban Planning, Arizona State University, Tempe, Arizona, United States of America; 2 Global Cities, The Nature Conservancy, Arlington, Virginia, United States of America; 3 Global Science, The Nature Conservancy, Fort Collins, Colorado, United States of America; 4 The Natural Capital Project, Stanford University, Stanford, California, United States of America; 5 Center for Environmental Systems Research, University of Kassel, Kassel, Germany; 6 Global Water, The Nature Conservancy, New York, New York, United States of America; 7 Tetra Tech Inc., Pasadena, California, United States of America; 8 Department of Engineering and Public Policy, Carnegie Mellon University, Pittsburgh, Pennsylvania, United States of America; 9 Latin America Region, The Nature Conservancy, Hollis, New Hampshire, United States of America; 10 Global Water, The Nature Conservancy, Seattle, Washington, United States of America; 11 Department of Geography, McGill University, Montreal, Canada; 12 Latin America Region, The Nature Conservancy, Curitiba, Brazil; University of Vermont, UNITED STATES

## Abstract

Governments, development banks, corporations, and nonprofits are increasingly considering the potential contribution of watershed conservation activities to secure clean water for cities and to reduce flood risk. These organizations, however, often lack decision-relevant, initial screening information across multiple cities to identify which specific city-watershed combinations present not only water-related risks but also potentially attractive opportunities for mitigation via natural infrastructure approaches. To address this need, this paper presents a novel methodology for a continental assessment of the potential for watershed conservation activities to improve surface drinking water quality and mitigate riverine and stormwater flood risks in 70 major cities across Latin America. We used publicly available geospatial data to analyze 887 associated watersheds. Water quality metrics assessed the potential for agricultural practices, afforestation, riparian buffers, and forest conservation to mitigate sediment and phosphorus loads. Flood reduction metrics analyzed the role of increasing infiltration, restoring riparian wetlands, and reducing connected impervious surface to mitigate riverine and stormwater floods for exposed urban populations. Cities were then categorized based on relative opportunity potential to reduce identified risks through watershed conservation activities. We find high opportunities for watershed activities to mitigate at least one of the risks in 42 cities, potentially benefiting 96 million people or around 60% of the urbanites living in the 70 largest cities in Latin America. We estimate water quality could be improved for 72 million people in 27 cities, riverine flood risk mitigated for 5 million people in 13 cities, and stormwater flooding mitigated for 44 million people in 14 cities. We identified five cities with the potential to simultaneously enhance water quality and mitigate flood risks, and in contrast, six cities where conservation efforts are unlikely to meaningfully mitigate either risk. Institutions investing in natural infrastructure to improve water security in Latin America can maximize their impact by focusing on specific watershed conservation activities either for cleaner drinking water or flood mitigation in cities identified in our analysis where these interventions are most likely to reduce risk.

## Introduction

Urban water security is the capacity of cities to “ensure access to adequate quantity and quality of water to sustain livelihoods, protect against pollution and disasters, and preserve ecosystems” [1]. Multiple studies have mapped water security risks at continental or global scales (e.g. [2–7]), providing useful insights for decision makers. While elucidating risks is an important component of informing decisions, these large geographic assessments often fail to identify where potential interventions, especially involving protection, restoration, or improved management of ecosystems, could most effectively mitigate water security risks [8]. Maintaining and improving “natural infrastructure”—that is, the conditions of the landscape that provide functions to mediate and regulate shocks and disturbances that threaten human well-being—can play an important role in water security [9].

Governments, development banks, corporations, and nonprofits are increasingly experimenting with natural infrastructure or landscape restoration [10] strategies for water security, recognizing their potential to be cost-effective and complementary to conventional built infrastructure [11]. To screen efficiently for opportunities, these institutions need standardized and credible, yet low cost and easily deployed methods to inform initial prioritization decisions to increase water security. Watershed intervention options, such as wetland restoration, forest thinning, agricultural best management practices, or installing urban bioswales, should be systematically compared and ranked for effectiveness across the geographic sphere of interest, which may include, for example, multiple locations at national or continental scales. One such group, with which we engaged as a test case through this project, is the Latin America Conservation Council (LACC), an entity of over 30 cross-sector leaders (e.g., Inter-American Development Bank, Development Bank of Latin America, The Coca-Cola Company) working to advance natural infrastructure solutions to major challenges in Latin America that benefit people and nature (http://laconservationcouncil.org/en). As water risks escalate throughout the region, LACC’s Working Group on Water Security has committed to using a natural infrastructure approach to help secure clean water supplies for at least 25 major cities in Latin America, thereby helping to enhance water security for cities inhabited by up to 100 million Latin Americans by 2025. Using scientific evidence to conduct an initial prioritization screen can help organizations like LACC with a broad geographic commitment focus their time and resources on places where they can more efficiently achieve their objectives.

No initial prioritization screen currently exists, nor do continental or global maps that identify what kind of natural infrastructure activities in which contributing watersheds could mitigate water security concerns for cities. The majority of previous work has focused solely on water supply or water quality, not flood risk. Efforts to assess the role of natural infrastructure across large regional or global geographies include a survey of 500 programs for natural infrastructure watershed investments [12,13], a global assessment of the opportunity to improve drinking water quality in 500 cities [14], and identification of protected areas under threat for watersheds upstream of dense populations [15]. Some existing tools (e.g., [16]) are available to prioritize natural infrastructure activities on a case-by-case basis. One of the main obstacles to scaling up natural infrastructure investments continues to be inadequate data, which inhibits comparison of the relative investment opportunities for multiple water security risks across a large number of watersheds [12,15,17,18].

Most literature focuses on natural infrastructure for just one watershed or case study, in part because existing methods to quantify the role of natural infrastructure for improving water quality [19], mitigating riverine floods [20], and reducing stormwater floods [21] are based on hydrologic models that must be calibrated for each specific watershed. These methods prove infeasible to implement at continental scales, because up to a dozen or more watersheds can be connected to drinking water or flood contributing areas for each city, each of which must be analyzed separately. When analyzing data for tens to hundreds of cities at a continental scale, the number of watershed models that require calibration and analysis grows exponentially. The large data and time requirements of existing methods thus poses substantial modeling barriers for continental-scale assessments and the ability to produce standardized information to allow regional actors to consistently compare conditions across cities.

This paper advances a new, relatively simple modeling approach that can be deployed with publicly available national and global datasets including land use, soil type, precipitation, elevation, population, fertilizer applications, water treatment costs, spatial flood exposure, and flood peak discharges, to conduct a high-level screening of where natural infrastructure activities are most (or least) relatively well positioned (primarily based on geophysical, ecological, and land use characteristics as a first determination of potential) to enhance water security for cities. This method further enables the comparison of relative effectiveness of natural infrastructure across a large number of cities at a national, continental, or potentially global scale. The analytical approach was developed and applied across 70 major cities in Latin America (including 887 watershed-based analyses) by a collaborative team of hydrologists, ecologists, engineers, economists, and Latin American conservation practitioners. This research was designed to provide actionable science to help inform the LACC’s water security strategy across Latin America, while also creating a method that could be transferred to comparable national or continental-scale decisions in other geographies and by other public, private, or civil sector actors.

Within this context, the analysis was designed to answer four research questions:

1. How do cities compare in terms of their relative potential for natural infrastructure to improve surface drinking water quality and mitigate riverine and stormwater flood risks?
2. How many people could potentially benefit across the region from improved water quality and reduced flood risks by targeting the source watersheds of high opportunity cities?
3. To what degree are the highest opportunity cities for improving surface drinking water quality aligned with high opportunity for mitigating flood risks?
4. How does a framework of targeting cities with the greatest opportunity for natural infrastructure strategies to mitigate risks differ from an approach based on targeting solely by level of risk?

## Materials and methods

The general approach, outlined in detail below, is to i) select cities and define urban extents, ii) map contributing watersheds—areas that drain into the city for flood risk and its surface water intake points for drinking water quality, also called “servicesheds”, iii) calculate water security risk and natural infrastructure mitigation opportunity scores for each serviceshed, iv) rank and categorize each city by combining risk and opportunity scores for associated servicesheds, and v) estimate the potential number of people in high, medium, and low opportunity categories across the continent. An important feature of our methodology is its evaluation of the relative effectiveness of watershed conservation activities to mitigate risks, rather than solely assessing risk itself. We chose this approach because of its expected importance to inform regional strategy decisions about where natural infrastructure is best positioned to deliver benefits. While risk is an important component for prioritization, conditions in servicesheds vary greatly in terms of whether risks can be effectively or minimally mitigated.

### City selection and defining urban extents

We analyzed the potential effectiveness of natural infrastructure interventions to contribute to securing clean drinking water supplies and mitigating flood risks for all urban agglomerations in Latin America with a population greater than 750,000 people based upon UN population estimates from 2010 [22]. Urban definitions followed the United Nation’s Population Division, which relied on self-reported urban areas defined by each country’s statistical office, most commonly based on administration, population density, and “functional” characteristics (e.g., paved streets) [22]. For urban extent, we used the Global Rural-Urban Mapping Project (GRUMP) urban extents based on nighttime light imagery and ancillary datasets [23,24]. For cities with overlapping GRUMP urban extents (e.g., the Colombian cities of Maracay, Caracas, and Valencia) or those not included in GRUMP (Havana, Cuba), we defined urban extents using the Global Administrative Database (GADM), which defines municipal urban administrative areas [25].

### Mapping servicesheds

Urban hydrologic ecosystem services are produced or provisioned in the contributing watershed upstream of the location where these services are consumed. In this paper, we refer to this spatial area as the “serviceshed” [26]. Cities source drinking water from a distinct set of watersheds, sometimes far from the urban area. In contrast, riverine flooding is generated in the contributing areas upstream (in elevation) from the urban area. Thus assessing natural infrastructure opportunity required spatial analysis defined by the source watershed and flood contributing areas connected to specific cities, which sometimes, but not always, overlap [27]. For each city, we analyzed three kinds of servicesheds:

1. “Drinking watersheds”, meaning the contributing area above drinking water withdrawal points from which surface water is delivered to the city. Withdrawal point data come from the City Water Map [14]. We focused on surface water sources, unlike other water sources such as groundwater, since watershed conservation activities to reduce sediment and phosphorus pollution are primarily targeted at surface water quality [28].
2. “Floodsheds”, meaning all contributing areas that drain into the urban extent.

**Fig 1:**
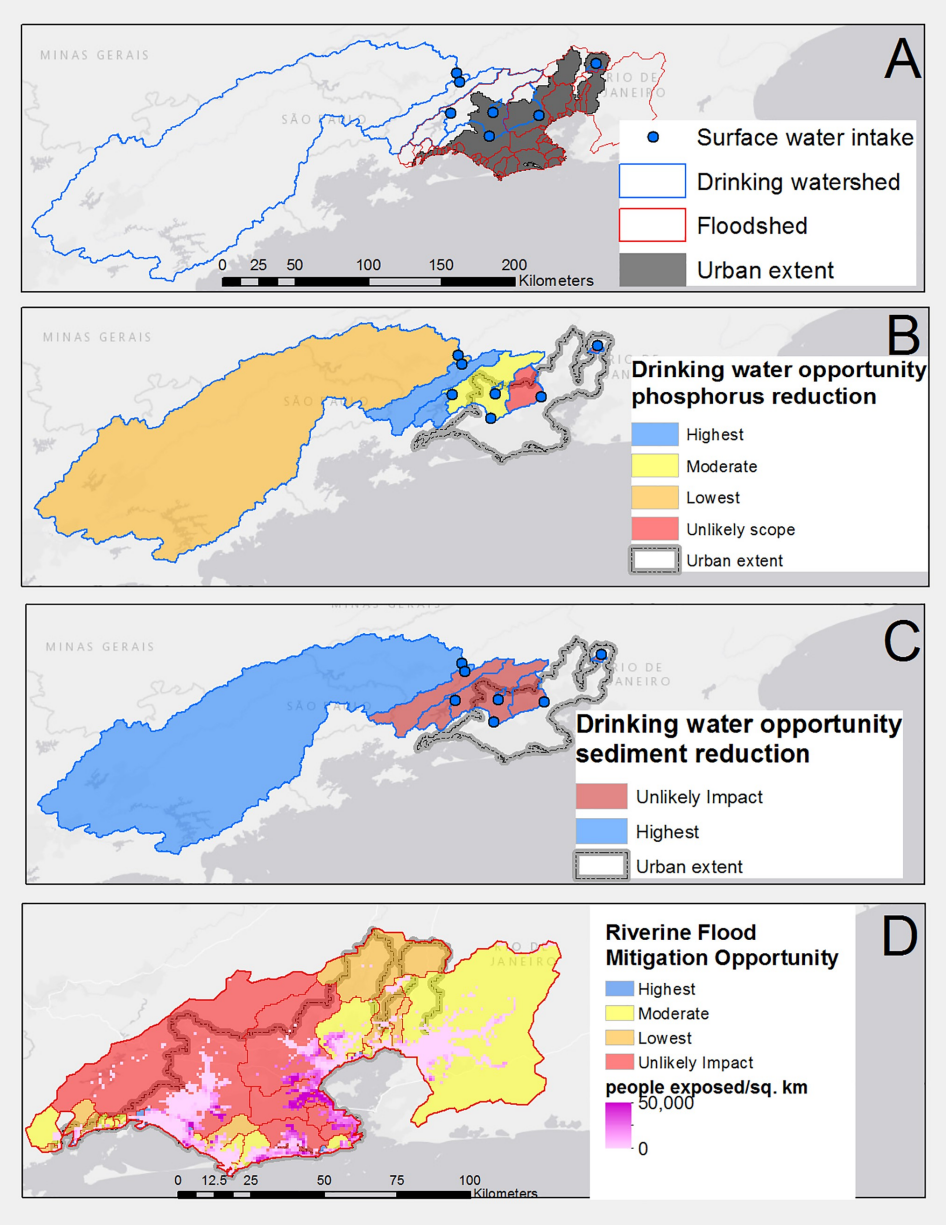
The spatial distribution of the city of Rio de Janeiro servicesheds for drinking water, riverine flood risk, and opportunities for mitigation with natural infrastructure. (A) Location of drinking watersheds (blue polygons) and floodsheds (red polygons). (B) Opportunity scores for each of Rio’s drinking watersheds related to the potential to reduce phosphorus loading via agricultural best management practices. (C) Opportunity scores for each of Rio’s drinking watersheds related to the potential to reduce sediment loading by restoring riparian areas. (D) Opportunity scores for each of Rio’s floodsheds related to riverine flood mitigation. The sewershed is congruent with the urban area, shown in grey in panel A, and outlined in gray in panel B and C.

“Sewersheds”, meaning the urban extent (as defined by GRUMP, and GADM, described above [23]) as a proxy for the generation of stormwater flooding generated by inadequate drainage networks and impervious surfaces. This approach was used because public data on sewer networks were unavailable for most cities in our Latin America analysis, which is also true for other regions (particularly cities in developing countries) where our method could be applied. Fig 1A shows an example of the location of the three servicesheds for the city of Rio de Janeiro. Based on our analysis, Rio has a moderate opportunity to mitigate stormwater flooding through natural infrastructure. Collectively, the details in this Fig 1 serve to illustrate key components of the analysis and results that were compiled for each of the 70 major cities in Latin America.

The 220 drinking watersheds were from the novel City Water Map database, developed through this and related projects, which maps surface intake coordinate points as reported by city water utilities [29], with contributing areas delineated from the HydroSHEDS 15 arc-second resolution flow accumulation and flow direction grids (i.e., with pixel sizes of approximately 250m at the equator) [30]. Similarly, a total of 776 floodsheds were identified by delineating all contributing areas upstream of the urban extent of each city. Based on expert opinion and media analysis, two corrections where made to the floodshed database for Porto Alegre and Manaus (both in Brazil), where the extended floodplains of rivers that flow outside the city boundaries could contribute to overbank flood risks in the urban areas. Finally, 70 sewersheds (the urban extent of each city) were analyzed to assess stormwater risk. A total of 887 distinct servicesheds (note some floodsheds and drinking watersheds overlap) across 70 urban areas were analyzed in this study.

Below, we describe the methodology to estimate the current population exposed to each risk (poor water quality, riverine flooding, and stormwater flooding) and the opportunity to mitigate these risks through watershed conservation activities that enhance water quality and flood regulation for each serviceshed.

### Modeling surface drinking water quality risks and opportunities for mitigation

#### Water quality risk in drinking servicesheds

The modeling for surface drinking water quality in source watersheds addressed sediments from sheet erosion and excess phosphorus (P) from agriculture, both of which are known to increase water treatment costs [14,29]. While other types of pollutants are also quite important for water managers (e.g., fecal coliform contamination), sediment and phosphorus are the pollutants most often targeted by and responsive to the watershed conservation activities considered in the analysis. Our metrics of surface water quality risk represent average annual sediment yield (tonnes/km^2^/yr) and P yield (kg/km^2^/yr). Pollutant yield was calculated with the available 15 arc-second resolution flow direction and accumulation grids for each contributing area at surface water intake points.

#### Sediment model

Global sediment loading was estimated using a modified version of the Universal Soil Loss Equation (see Table 1). The *R*-factor is rainfall erosivity, and a global map of this factor for current climate was obtained from the website climatewizard.org [31]. The *K*-factor is soil erodibility, which was estimated by converting the soil texture values found in the Harmonized World Soils Database to *K* values using the methodology of Roose [32]. The *LS*-factor is the slope-length, and it was estimated using the HydroSHEDS 15-arc second digital elevation model (DEM) following the methodology of the InVEST Sediment Retention Model’s calculation of the *LS*-factor [33]. The crop and practice (*CP*) factors relate to land cover and land use practices, and average values for different land use types were taken from the Spreadsheet Tool for Estimating Pollutant Load (STEPL) model [34] and the Watershed Treatment Model [35]. Our global land cover map was GlobCover 2009 available at 10-arc second resolution (~300m) [36], reclassified into six categories: Agricultural, Grassland/Pasture, Forest, Barren, Urban, and Water/Other (see [14] for reclassification details). We used a global average value for *CP* for all agricultural pixels and did not model differences in crop types or practices.

**Table 1:** Indicators used for surface drinking water quality risk and opportunity analysis in drinking watersheds.

Risk or Opportunity	Indicator	Method	Data
**Risk** Indicator * population * % surface water	Sediment Load (tonnes/km^2^/yr)	USLE (Universal Soil Loss Equation) Sediment_Load_ = RKLSCP R is rainfall erosivity, K soil erodibility, LS slope-length, CP is crop and practice factors.	R- Girvetz et al 2009. K- Roose et al. (1996) from FAO (2007) LS- HydroSHEDS CP- STEPL(Spreadsheet Tool for the Estimation of Pollutant Load), Water Treatment Model and GlobCover 2009
	Phosphorus Load (kg/km^2^/yr)	Export coefficient method [37], SPARROW delivery ratios by land use [38]	Global Fertilizer and Manure version 1 [39] and GlobCover 2009
**Opportunity** *Opportunity was calculated for each drinking watershed by estimating how many hectares for each of the 5 activities would be required to achieve a 10% reduction in sediment and phosphorus loading*. *A smaller number of hectares required to reach 10% reduction target means a higher opportunity*.	Agricultural Best Management Practices	All agricultural land Sediment: 72% reduction Phosphorus: 77% reduction [14]	Based on average results for implementing cover crops [40,41]
	Riparian Habitat Buffer	10m buffer on either side of rivers, as defined by HydroSHEDS. All agricultural land in this buffer is considered as candidate area for this strategy. Sediment: 86% reduction Phosphorus: 71.9% reduction [14]	Based on average results for implementing 10 m buffers [42]
	Pastureland Reforestation	Current grassland or pasture pixels that are in natural forested areas, as defined in WWF ecoregions [43]. Sediment: Change in CP factor from grassland. to forest Phosphorus: Change in export from grassland to forest.	See STEPL [34] and Water Treatment Model for crop and practice factors [35].
	Forest fuel reduction	Current natural forested land, as defined in WWF ecoregions as areas ecologically apt to be forested that currently have forest cover. The expected increase in pollutant load was defined as the probability of forest fire times the change in pollutant load if that occurs. Probability of forest fire calculated from Global Fire Emissions Database, version 4 [44]. Forest thinning reduces probability of a severe fire by 70%, based on review paper [45]. If fire occurs, then changes in sediment (but P not considered) calculated as: change in CP factor from natural land cover to barren.	Fuel management effectiveness average based on Martinson and Omi [45]
	Forest Protection	Current forested pixels that are in their natural area, as defined in WWF ecoregions. The expected increase in pollutant load, defined as probability of habitat loss times the change in pollutant load if that occurs. Probability of natural habitat loss without action calculated as biome averages between GlobCover images (2005 and 2009). If that loss occurs, then changes calculated as: Sediment: Change in CP factor from natural land cover to agricultural or ranchland. Phosphorus: Change in export from natural land cover to agricultural or ranchland.	See STEPL [34] and Water Treatment Model for crop and practice factors [35].

In order to calibrate the sediment model, our estimated sediment loading for each source watershed in the United States was compared with the SPARROW (SPAtially Referenced Regressions on Watershed attributes) dataset [46], which is an empirically-based estimate of loading calculated from thousands of direct stream measurements in the United States. These measurements are used to calibrate estimates of sediment loading based on sediment source, land use, and stream channel characteristics. Correlations between our loading estimates and those in the SPARROW dataset were generally strong (R ~0.8). Using a log-log linear regression, we adjusted our predicted estimate of loading to match SPARROW measurements of loading (p < .0001).

#### Phosphorus model

Phosphorus (P) loading was estimated using an export coefficient approach, where each land use/land cover type is modeled to export a certain average annual amount of P from the pixel. For forest, barren, urban, and water/other, the export coefficient was constant, using average values for different land cover types taken from the STEPL model and the Water Treatment Model. For agriculture and grassland/pasture, we based P export on the global grids of the Global Fertilizer and Manure (GFM) dataset, Version 1 [39]. Agricultural land was assumed to have both manure and fertilizer applied at the rates specified by the GFM, while grassland/pasture was assumed to have only manure applied at the rates specified by the GFM. The nutrient utilization efficiency (NUE; the fraction taken up by plants or soil, and not exported) was estimated using continental data for NUE [47].

As with sediment, our estimated P loading for each source watershed in the United States was compared with the SPARROW dataset. Correlations between our phosphorus loading estimates and those in the SPARROW dataset were generally strong (R ~0.8). We calibrated our results to the SPARROW estimates using a log-log linear regression (p < .0001).

#### Water quality opportunity in source watersheds

To analyze the potential opportunity of watershed conservation activities to reduce pollutants in source watersheds, we used information on changes in pollutant load in our calculations. Pollutant concentration, which has economic impacts on the operations and maintenance (O&M) costs of water treatment plants (WTPs), is calculated as load divided by river flow [29,48]. Note that in this study we quantify how changes in pollutant loading in a given watershed affect water quality. Specifically, we modelled the proportional reduction in pollutant loading from the baseline, defined as the load after conservation action divided by the load before conservation action. We quantified the potential of five commonly used source watershed conservation activities to cause a reduction in pollutant loadings for water that feeds into city drinking water supplies: (1) agricultural best management practices (BMPs), (2) riparian habitat buffers, (3) pastureland reforestation on existing grassland landcovers in forest biomes, (4) forest protection, and (5) forest fuel reduction.

Ideally, we would have been able to model change not in pollutant loading, but in pollutant concentration, but constructing a full hydrologic model of water flows was beyond the scope of this paper. However, note that if the effect of the conservation activity on flow is negligible, the proportional change in pollutant loading is approximately the same as the proportional change in concentration, because the flow terms cancel out (Eq 1).

ΔConcentration=LoadafterFlowLoadbeforeFlow Eq 1

We acknowledge that some conservation activities have an effect on flow [49], particularly in certain ecosystem types, so what we have modelled (change in pollutant loading) will only be approximately equivalent to the change in pollutant concentration.

We used estimates of average percent reduction in pollutants from the literature to calculate how implementation of each activity would reduce sediment and phosphorus loadings (Table 1, Methods column) [14]. Each source watershed contains many pixels, so there are multiple places where a practice could be performed. The median or average expected benefit from a practice in a watershed may not be the most meaningful metric since conservation actions will likely focus on sites where it will yield the greatest return. We thus calculated the number of hectares requiring conservation actions to achieve a nominal 10% reduction in the pollutant, assuming watershed activities started at the pixels with the highest return for agricultural BMPs, riparian habitat buffers, and pastureland reforestation. Based on a statistical analysis of empirical data (see McDonald and Shemie [14] for details), a 10 percent reduction in pollutant is worth around a 5 percent reduction in O&M costs for a conventional WTP processing city water. Thus, a 10% reduction in a pollutant is likely to generate significant cost savings.

Forest protection (in areas at risk of conversion) and forest fuel reduction reduce a future risk of increased sediment or nutrient loading by preventing deforestation and forest fires. For these two activities, we calculated the amount of land required to reduce future pollutant loading by 10 percent. Future loading is defined as the current baseline pollutant load plus the expected future increase in loading based on the probability of future deforestation or fire (see Table 1 for calculation method).

#### Identifying opportunity at the city scale

For each watershed, we calculated how many hectares of each activity were required to reduce sediment or phosphorus by at least 10%. We then determined which of the five strategies was most efficient, in terms of where conservation of the least amount of hectares produced the greatest potential improvement in drinking water quality. Cities requiring less than 1,000 ha of watershed conservation activities to achieve the minimum 10% target reduction in either sediment or phosphorus were ranked as having the ‘highest’ potential of investment in natural infrastructure; cities requiring 1,000–10,000 ha were ranked as ‘moderate’; and cities requiring more than 10,000 ha were ranked as ‘lowest’ (see Fig 1B and 1C to see an example of how drinking water servicesheds were categorized for Rio de Janeiro). These breaks roughly divided source watersheds globally into three equal sized groups [14]. Finally, based upon our modeling, cities that could not hit the 10% reduction target for either pollutant, or that do not source the majority of their water supply from watersheds that can be helped by the activities studied (e.g., all cities that depend on more than 50% on groundwater), were ranked as ‘unlikely scope’. For instance, if there is not sufficient land in a watershed where it is possible to do reforestation, then this conservation activity may be unable to reduce sediment load by 10%.

Note also that the calculation is a city-level average ranking, with each source weighted by the volume of water it contributes to a city’s supply as reported in the City Water Map [14]. Fifty-six of the 70 cities analyzed have more than one individual source watershed as part of their overall drinking watershed. These individual source watersheds may have high investment potential even if the overall city ranking is low for its drinking watershed.

### Modeling riverine floodshed risks and opportunities for mitigation

#### Riverine flood risk indicators

Riverine flood exposure was estimated with the global map of inundation extents GIEMS-D15 [50]. This raster dataset at 15 arc-second pixel resolution (approximately 500 m at the equator) identifies flood risk at three temporal states of inundation: mean annual minimum extent (representing the dry season extent), mean annual maximum extent (representing the wet season extent), and the historic maximum extent. We estimated the number of people exposed to flooding for each city floodshed by summing the total population (from Landscan [51]) within its urban area in the mean annual maximum inundation extent as reported by GIEMS-D15.

#### Riverine flood mitigation opportunity indicators

Vegetation functions as natural infrastructure for riverine flood mitigation by mitigating the timing, duration, depth, velocity, or location of flood peaks that may lead to increased loss to lives or livelihoods during an extreme rainfall event. Opportunities to mitigate riverine flooding were assessed by estimating both the area of eligible land where natural infrastructure activities could be performed, which we refer to as “scope of intervention”, and the likelihood that these interventions could reduce flood peaks given the biophysical conditions of each watershed, which we refer to as “watershed response sensitivity” (Eq 2).

Opportunity=WatershedResponseSensitivity+PotentialScopeofIntervention Eq 2

The biophysical factors that condition watershed response sensitivity to flooding were selected from a limited literature review, expert opinion, and assumptions from geomorphology theory. Factors include: watershed shape (roundness) [52], slope [52], size [53], drainage density [54], and sensitivity of flooding due to standardized changes in peak discharges and inundation areas. These five indicators are combined as defined in Eq 3.

Theoretical assumptions and expert review inform assumptions due to scant literature on empirical studies that review or explicitly compare watershed flood response to land cover change across these factors for large numbers of watersheds. This is in part due to a bias in hydrology and land use change studies to focus on one watershed over time, or pairwise comparisons of just two basins [55]. Note the possibility that the importance of each factor in predicting flooding (which we have assumed to be weighted equally) varies with flood frequency, however, no available data exists to suggest an improved weighting scheme across the continent.

WatershedResponseSensitivity=Shape+Slope+DrainageDensity+Size+FloodSensitivity Eq 3

The first four factors were calculated using digital elevation data, watershed delineation in ArcGIS, and stream network data (see Table 2). Watershed shape is calculated using the Gravelius Index [56]. We assume that rounder watersheds, which geomorphology theory indicates having higher peak flood discharge volume [57], will be more sensitive to relative reductions in flood peak flow with land cover changes. Lower sloped watersheds are theoretically less “flashy” and have longer rises to peak flood. A relatively longer rise to peak flood due to topographic conditions means larger volumes of water have an opportunity to infiltrate over the land surface on a longer time horizon. We thus assume floods in lower slope watersheds are more sensitive to changes in land cover. These assumptions are supported by at least one empirical study [52]. High drainage density watersheds tend to be “flashy” and more prone to flooding, supported by empirical reviews [58]. We assume the proposed watershed conservation activities to disconnect impervious surface areas to the drainage network via riparian buffers could theoretically significantly slow down flood peaks in high drainage density watersheds. While this is supported theoretically and by expert opinion, it has yet to be demonstrated empirically across multiple watersheds. We assume smaller watersheds can be more effective for restoration, as a smaller investment in land area is needed to change the same percentage of the watershed area, which is supported by at least one empirical study [59].

**Table 2:** Indicators used for riverine floodshed risk and opportunity analysis.

Risk or Opportunity	Opportunity category	Indicator	Method	Data
***Risk***		Number of people exposed to annual flooding	Sum of population in the floodplain per floodshed	Landscan 2012 (population), GIEMS-D15 (floodplains) [50]
***Opportunity***^1^	Watershed Response Sensitivity (Eq 3)	Watershed shape	Roundness [52] as measured by the Gravelius Index (Gravelius 1914), G = 0.2841*P/A^.5, where *P* is perimeter and *A* is area. A value of 1 represents a perfectly round watershed.	HydroSheds 15-sec drainage basins
		Slope*	Average Watershed Slope in degrees, using the SRTM Digital Elevation Model to estimate slope in degrees with the Jenness DEM Surface Tool [60]	SRTM Digital Elevation
		Size*	Total area in km^2^ [59]	HydroSHEDS 15-sec drainage basins
		Drainage density	Total channel length/basin area (km/km^2^) [54]	HydroSheds 15-sec river network
		Flood discharge sensitivity	$Flood-Q\;Sensitivity=\frac{{(I}_{12}-I_{11})/A_{city}}{{(Q}_{12}-Q_{11})/MQ}$ where *I*_*x*_ is the inundated area (in km^2^) within the city limits in month *x*, *A*_*city*_ is the area of the city (in km^2^), *Q*_*x*_ is the average discharge in month *x* and *MQ* is the long-term average discharge. 12 represents the highest and 11 the second highest month of discharge for each basin.	Inundation data:GIEMS-D15(the authors of GIEMS-D15 provided us with a version that includes monthly inundation extents) Discharge Data: Estimates from the global hydrological model WaterGAP downscaled to the HydroSHEDS resolution using geostatistical approaches [61,62]
	Potential Scope of Intervention (Eq 4)	Preserving infiltration	Estimated average basin curve number (CN) [63]	Soils: FAO (2007) [64], Land use: GlobCover (2009) [36]
		Increasing infiltration	Current CN minus Lowest Potential CN if all non-urban land is restored to natural	Soils: FAO (2007), Land use/cover: GlobCover (2009)
		Preserving Effective Pervious	% of total natural area directly connected to stream network of the basin	HydroSHEDS, Globcover (2009)
		Disconnecting effective impervious area*	Riparian area in km^2^ required around streams to disconnect urban area with a 500 meter buffer [65]	HydroSHEDS, GlobCover (2009)
		Preserving wetland storage	Percent of watershed with natural riparian wetlands intact. Wetlands defined by perennial flooded area in GIEMS-D15, “natural” defined as areas not in pasture/ grassland in WWF forest biomes, urban or agricultural land use categories in GlobCover (2009)	GlobCover (2009), GIEMS-D15
		Increasing wetland storage	Potential increase in wetland area if “recovered” wetland (areas that could be wetland but are currently bare, agricultural, or pastoral) is added to the area of “existing” wetlands (wetlands currently in “natural” land covers) to estimate the total possible wetland area as a percent of each watershed. The potential percent increase in wetland storage represents the gain if all wetlands are restored. [66]	Globcover (2009), GIEMS-D15

^1^All opportunity indices were normalized and then added. Indices were inverted when a lower score was “better”. These are marked with an “*”.

Flood discharge sensitivity (for equation see Table 2) is a new index based on the assumption that watersheds where small changes in relative discharge lead to great changes in relative flood area are sensitive to discharge alterations and thus interventions could have the greatest impact.

To preserve distribution of these continuous variables, each metric was scaled from 0–1 by removing the minimum value and dividing by the maximum value for that variable, with a value of zero representing the least desirable condition (e.g., no change in flood risk even with high changes in discharge, indicating that conservation efforts designed to reduce flood peak discharges are unlikely to influence flood extents) and a value of one representing the most desirable condition (e.g., smallest area, because a relatively small conservation investment can represent a large change in the proportion of restored land use in the watershed, and it is the change in proportion that tends to most influence hydrologic function for flood risk [67]). This relative ranking was used in lieu of thresholds or data binning, as we found no empirical basis in scientific literature for thresholds. Scaling to one was preferred over using z-scores or standard deviations to normalize each variable in order to preserve the lowest values for each indicator at zero. Scaling to one also preserves outlier watersheds with very high sensitivity for conservation action to be taken into consideration when considering city-level floodshed scores (which is a summarized score of opportunities across all flood watersheds). We chose to preserve raw data for watershed level indicators rather than bin them, especially due to the limited empirical literature relative to the other water security risks studies. The watershed sensitivity index was computed on a score from 0–5 by adding each of the five indicators.

There are two main types of flood mitigation activities: (i) conservation to protect existing lands at risk of conversion to another land cover, and (ii) restoration activities to change an existing land cover (usually pasture/grassland in what ecologically would be a forest biome or agriculture) to natural vegetation. Flood risk is expected to double by 2030 due to both climate and demographic changes[6]. Therefore preserving existing natural infrastructure may be just as important in mitigating future flood risk as restoration activity to retain existing infiltration capacity. The assessment of the potential scope of watershed intervention to reduce flood peaks was based on six indicators that represent availability of restoration and protection activities that mitigate flooding: preserving or increasing infiltration [68], preserving effective pervious area, disconnecting effective impervious area [69,70], and preserving or increasing wetland and floodplain storage (see Table 2). These are combined in Eq 4 (note use of abbreviations: Pres. = preserving; Incr. = increasing; Discon. = disconnecting).

InterventionScope=Pres.infiltration+Incr.infiltration+Pres.perviousarea+Discon.imperviousarea+Pres.wetlandstorage+Incr.wetlandstorage Eq 4

Preserving infiltration was estimated using the “Curve Number” (CN) for each watershed. The National Resources Conservation Service (NRCS) curve number (CN) is an empirical parameter developed through experiments by the USDA (United States Department of Agriculture) to estimate the ratio of runoff to rainfall excess for specific combinations of land use, soil type, and antecedent soil moisture condition [63]. Lower average CN values given current land cover indicate higher infiltration capacity. Extant land covers could be preserved to protect this ecosystem service. The second intervention scope indicator estimates restoration potential. We first calculated the lowest potential CN (for the requisite land use-soil type combination) for “restorable” non-urban land uses (e.g., barren, deforested areas, agriculture, pasture in forest biomes). Several studies have shown the capacity of degraded land to regain infiltration capacity, even for working landscapes such as farms and plantations [71,72]. However, the ability to restore infiltration properties is complex, and restoration tends to most effectively increase infiltration on natural permeable soils [68]. We then calculated the percent decrease in CN potential assuming these non-urban areas could be restored.

Impervious area buffer is calculated as the percent of the riparian area in a 500 m buffer around the stream that is not in urban land use. Riparian areas around a stream are important to keep undeveloped to reduce effective urban areas and allow natural channel and geomorphic adjustments. These areas can be an important buffer barrier between high velocity urban runoff and the stream [69].

“Effective” impervious area refers to urban areas spatially connected to perennial streams. Disconnecting this effective impervious area would require reclaiming a 500 m buffer area around the river anywhere that urban land touches a perennial stream. This buffer prevents urban runoff from entering the stream directly as overland flow, reducing the flow velocity via the rougher friction of vegetation [69]. This metric first estimates the buffer area (in km^2^) required to disconnect all effective total impervious area for each basin. This required buffer area is then divided by the total effective impervious area of the basin. The resulting ratio is an efficiency metric, where lower values represent less effort (fewer km^2^) required to disconnect each km^2^ of urban land from the stream.

Preserving floodplain storage was calculated as the percent riparian wetland in a watershed according to the minimum annual flooded areas from GIEMS-D15 [50]. These perennially flooded areas are assumed to provide persistent floodplain or wetland habitat. Increasing floodplain storage was calculated as the potential increase in riparian wetland area as a percentage of the watershed if riparian wetland were restored on existing pasture and agricultural land that floods on average at least once annually (according to GIEMS-D15 data). Wetlands can store large amounts of water and play an important role in flood mitigation [66]. Similar to the watershed response sensitivity index, these metrics were computed for each of the six interventions and were normalized for each watershed between 0 (lowest potential) and 1 (highest potential).

#### Identifying opportunity at the city-scale

Each watershed was scored for opportunity by adding together all eleven indicators (response sensitivity plus scope of intervention metrics listed in Table 2). The opportunity scores are normally distributed across watersheds, despite some “outlier” watersheds in individual metrics for scope or sensitivity. Thus, the opportunity index methodology presented is relatively robust to outlier watersheds. See Fig 1C as an example of opportunity categories for each of Rio de Janeiro’s floodsheds. The final city score was calculated as a weighted average based on the estimated number of people exposed to flooding in each individual floodshed (see Eq 5).

$$City\;Score=\frac{\sum_{J=1}^{n}{(\#flood\;exposed\;population\;per\;basin}_{J}\;x\;{flood\;watershed\;opportunity\;score}_{J)}}{\sum_{J=1}^{n}{\#flood\;exposed\;population\;per\;basin}_{J}}$$ Eq 5

Absent literature that helped define thresholds, cities were then binned by quintile scores and qualified as having highest opportunity (top quintile), moderate opportunity (second and third quintile), lowest opportunity (fourth) and unlikely scope (fifth quintile). Cities in each of these categories were then sorted by the number of people exposed to flooding, such that cities with more people at risk were ranked first in their respective opportunity category.

### Modeling stormwater floodshed risks and opportunities for mitigation

To assess the relative potential of natural infrastructure to mitigate stormwater for each city, indicators representing relative stormwater risks and opportunity for natural infrastructure to mitigate risks were calculated for each sewershed (Table 3) and added together (Eq 6).

StormwaterRankScore=StormwaterRisk+UrbanNaturalInfrastructureOpportunity Eq 6

**Table 3:** Indicators used for stormwater flooding.

Risk or Opportunity	Indicator	Method	Data
**Risk**	Relative Storm Intensity	# of annual dry days/average annual mm precipitation. Cities ranked based on this indicator in descending order and divided into quartiles to receive a score (4,3,2,1) for their respective quartile.	Climate Research Unit at East Anglia (2005) CRU CL v. 2.0 [73].
	Soil Permeability	USDA hydrologic soil group type (a-d) given a score (1–4) from lowest to highest permeability (type a = 1, type b = 2, type c = 3, type d = 4). City scores are area weighted composites based on FAO spatial data.	FAO (2007)
**Opportunity**	Percent Open Space	Binary classification via thresholding of band 5 Landsat imagery. Non-impervious land considered open space. Cities with 40–50% open space are scored highest. See scores in Table 4 and in the text description.	Calibrated global imagery from Hansen et al. (2013) data set analyzed in Google Earth Engine
	Distribution of Open Space	Nearest neighbor spatial statistics calculated for each vegetated patch in a city. Cities with value greater than 0 have dispersed open spaces (preferred) and cities with less than 0 have a more “clustered” open space. Evenly distributed open space indicates the likelihood of mitigating potential stormwater flooding at the city scale.	Estimated from percent open space (see above)
	Average City Slope	Slope 2–4% scored 4, 4–6% scored 3, 6–8% scored 2, 8–10% scored 1. Slope outside this range scored 0. Natural infrastructure for flood mitigation is easier to implement on lower, but not flat, slopes.	SRTM 90-m DEM [74]

**Table 4:** Percent open space scoring. Pixels classified as built up are scored as 80% impervious, thus a cities impervious area estimation is ~20% lower than the % urban space estimation.

% Open Space	% Built up Space	% Impervious Area	Score
87.5	12.5	0–10	0
75	25	10–20	1
62.5	37.5	20–30	2
50	50	30–40	3
37.5	62.5	40–50	4
25	75	50–60	3
12.5	87.5	60–70	2
0	100	70–80	1

#### Stormwater flood risk indicators

Two indicators represent the risk of a stormwater flood hazard (Eq 7), precipitation intensity and soil permeability. Higher precipitation intensity and lower soil permeability can lead to overwhelmed drainage systems and localized flooding.

StormwaterRisk=RelativePrecip.IntensityScore+SoilScore Eq 7

Relative precipitation intensity is calculated as the average number of dry days divided by the average annual precipitation derived from global observations [73] from 1961–1990. This higher relative storm intensity indicates cities that are more likely to experience frequent storms that can lead to overwhelmed drainage systems and localized flooding. This index was computed for each urban extent and cities were scored by quartile from 1–4, with a value of 1 being cities with the least intense storms and a value of 4 being cities with the most intense storms. In addition to precipitation, soil type [64] is another flood risk indicator. If a city is located in areas that are typical of clay soils, there is a higher likelihood that localized flooding issues will occur. The percent of USDA hydrologic soil groups was calculated for each city and scored using a range of 1–4 with higher scores indicative of lower permeability soils (see Table 3). A weighted area soil score was calculated for each city.

#### Stormwater flood mitigation opportunity indicators

Three indicators represent the opportunity for natural infrastructure placement, as shown in Eq 8. Reviews of natural infrastructure performance for stormwater indicate most studies compare site rather than catchment scales [75]. We thus relied on expert opinion and experience with natural infrastructure installation from a leading engineering firm Tetra Tech, Inc (included by R.D., author on this paper) in this initial study to indicate scores for each of these three indicators. Sensitivity analysis of each indicator is left for future research and beyond the scope of this initial assessment.

UrbanNaturalInfrastuctureOpportunity=percentopenspacewithincity+spatialdistributionofopenspacethroughoutdevelopedareas+averageslopeofthecity Eq 8

Percent open space was calculated as percent of the city that was not covered by impervious surfaces. Globally available urban cover data sets were not of sufficiently high resolution at the time of this analysis (2014) to distinguish between open green space and impervious area for Latin American cities. Thus impervious area estimates were derived by classifying pixels below a threshold of 5 in modified Band 5 (short-wave infrared) of the Landsat 7 ETM+ 30 m resolution multispectral image composite from 2012 provided by Hansen et al. (2013) via Google Earth Engine [76] as highly built up area, and pixels above this threshold as non built up area. Other studies comparing land use to impervious area [77] list a range of 35–70% impervious for urban land uses but can stretch over 90% for areas within Manhattan, New York City, USA. We assumed any “built up” pixels to be 80% impervious. Non built up areas were considered “open space.” Cities with 40–50% impervious were scored highest (4), and higher or lower percentages of open spaces were scored from 1–3 as they departed from this mid-range imperviousness. Engineering experience indicated that large areas with somewhat less than half its land cover as impervious are high enough to pose a problem, yet with sufficient open space to still leave sufficient potential to site natural infrastructure.

The next indicator, distribution of open space, was calculated by using nearest neighbor spatial statistics, which take the average distance between all pervious surfaces divided by the total number of pervious surface patches Evenly distributed open space indicates higher likelihood of mitigating potential stormwater flooding. The resultant level of dispersion was binned using quartile-based thresholds determined from across all cities, and scored 1–4 with 4 being the highest level of dispersion (most distributed open space). Finally, average city slope was calculated, assuming it is easier to implement natural infrastructure on lower (but not flat) sloped land (see Table 3 for scoring).

#### Identifying opportunity at the city scale

Each of the indicators described above was scored 1–4 based on experience from Tetra Tech (through author R.D.) and a literature review, with 4 being the most desirable category of each indicator to mitigate stormwater flooding. These 5 unweighted indicators were then added together for each city (see Eq 7). Cities were ranked by highest score. As for the riverine flood mitigation opportunity index, the top quintile cities were selected as the highest opportunity, the second and third quintiles as moderate, the fourth as lowest, and the fifth as unlikely impact. Cities were sorted by highest total urban population within each of these opportunity categories. In lieu of a stormwater flood risk continental map, we assumed that all urban populations could benefit from decreased stormwater flooding, which can disrupt transportation, electricity, and other services and property for entire urban populations.

### Estimating potential benefiaries for natural infrastructure across servicesheds at the city scale

The metrics described above for natural infrastructure opportunity to mitigate risks for drinking water quality, riverine flooding, and stormwater flooding were calculated for each ‘serviceshed’ (summarized in Table 5). Recall that each serviceshed may be comprised of several individual watersheds or contributing areas that supply the demand for that service. A single city score for riverine flood mitigation or drinking water often required a method to combine many individual watershed level scores for each city. City scores were calculated using a weighted average of individual watershed scores for drinking water (weighted by the proportion of water supply volume it contributed to the city) and riverine flood mitigation (weighted by exposed flood population, see Eq 5). The stormwater “serviceshed” for each city is not comprised of many individual watersheds, as the ecosystem service supply area is identical to the urban extent for that city. In contrast to riverine and drinking water analyses which required analysis of many individual watersheds, no additional compilation of individual watersheds (e.g. a weighted average) was needed to generate a composite stormwater opportunity city score.

**Table 5:** Summary of risk and opportunity metrics included in the prioritization analysis for surface drinking water quality, riverine flooding, and stormwater flooding.

		Surface drinking water quality	Riverine flooding	Stormwater flooding
**Risk**	***Definition***	Urban population that relies on surface drinking water	Urban population in the floodplain	Urban population in cities with stormwater flood risk
	***Indicators***	Annual sediment load, annual phosphorus load, number of people relying on source drinking water	Number of people exposed to annual flooding	Total Urban Population*
**Opportunity**	***Definition***	Number of hectares required to achieve a 10% reduction in sediment and nutrient loading	Watershed response sensitivity and scope of conservation action	Scope of conservation intervention in the urban area and potential effectiveness
	***Indicators***	Hectares related to specific conservation activities: agricultural best management practices, riparian habitat buffer, pastureland reforestation, forest fuel reduction, forest protection	Shape, slope, size, drainage density, flood discharge sensitivity, preserving and increasing infiltration, preserving effective pervious area, disconnecting effective impervious area, preserving and increasing wetland storage	Percent open space, distribution of open space, average city slope

*Given inadequate continental scale data on stormwater flood risk exposure, we assume all urban populations may experience flood risk. We did a relative ranking of cities which may be more exposed to these risks due to more intense storms or less permeable soil types, but it was not possible to estimate the population differences across cities.

In most cases, a single city has multiple servicesheds, some of which uniquely influence each type of water security risk (Fig 1). Thus, no water security opportunity score that combined mitigation for all three risks was generated. Instead, cities were ranked for their relative opportunity for natural infrastructure to individually mitigate each of the three risks. We calculated the number of beneficiaries for each risk mitigation per city by summing the number of people “exposed” to that risk using the metrics in Table 5. For drinking water, the number of potential beneficiaries per basin was calculated based on the percentage of a city’s drinking water withdrawn from each surface intake source watershed, multiplied by the city’s population. The number of potential beneficiaries per city subject to riverine floods is the total number of people exposed to flooding.

We assumed all urban residents (e.g., the total city population) could be subject to stormwater flooding either directly in their own homes or communities, or indirectly, for example, through transportation delays due to flooded roadways. While cities were ranked for relative stormwater risk based on the frequency of intense storms and soil permeability (Eq 7) we did not use these metrics to rescale or alter our estimated number of people exposed to stormwater floods due to lack of evidence. That is, we do not know how many fewer people are exposed to stormwater flooding based on climate or soil variables, because no baseline for stormwater exposure exists. In contrast, available data exists estimating population reliance on both drinking water sources and flood risk exposure.

Developing a city-level score of each natural infrastructure mitigation opportunity enabled consistent reporting on standardized spatial units to facilitate the city-to-city ranking. It is important to emphasize these city scores were not used to rank cities ordinally (i.e., 1–70) for each type of natural infrastructure opportunity, because using global data to assess hundreds of watersheds at the continental scale is unlikely to support such a specific ranking. Instead, we classify each city into broader categories of highest, moderate, lowest, and unlikely impact category and use these relative rankings to report the results.

We define the highest opportunity cities–for the purpose of prioritization–as those where natural infrastructure can make the greatest impact, whereas the lowest opportunity cities are those where natural infrastructure is least likely to be effective and there is potentially a need for greater relative use of built infrastructure.

### Estimated urban population in each opportunity category

Urban populations that could benefit from reduced flood exposure or improved water quality for the highest, moderate, and lowest opportunity categories for each of the three risks and mitigation opportunities were estimated in each serviceshed. We then estimated the total Latin American urban population that could benefit from any of the three water security risks in each opportunity category, ensuring not to double count by using the highest population number (population using drinking water from surface source, population exposed to riverine floods, or the total urban population in the case of storm water) when a city was ranked at the same opportunity category for more than one type of water security risk. For example, when calculating the number of people who could benefit from increased water security in the “highest” category, if an estimated 70,000 people were in a city that is considered in the “highest” category for water quality improvements, and this same city also ranked “highest” for flood mitigation that could benefit 10,000 people, we estimated that city had and overall 70,000 residents overall that could benefit from natural infrastructure, instead of adding beneficiaries from both types of water risk mitigation to obtain 80,000 beneficiaries. This is because the urban residents who live in a floodplain may be the same residents who could enjoy improved drinking water, and should not be counted twice.

For some cities, drinking watersheds overlap spatially with floodsheds. In these contexts, a set of well-designed conservation activities in the same watersheds can effectively mitigate risks for more than one water issue at a time–often referred to in the ecosystem services literature as co-benefits [78]. To evaluate the potential for co-benefits, cities in which implementing natural infrastructure activities to improve surface water quality could also mitigate riverine flooding were identified by the spatial overlap of high opportunity to enhance drinking water and high or very high opportunity to mitigate riverine flooding within the same watersheds. Co-benefits between stormwater flood mitigation and other water security metrics were not calculated, because the location and type of watershed conservation activities required for stormwater flood mitigation are very different from location and type of activities required to enhance water quality or mitigate riverine flood peaks. Stormwater mitigation conservation activities occur in downstream urban areas to mitigate pressure on sewer systems, while riverine flood and drinking water activities often occur upstream, in agricultural areas, where activities to enhance water quality or mitigate riverine flood peaks are likely to have greater impact.

## Results

**Fig 2:**
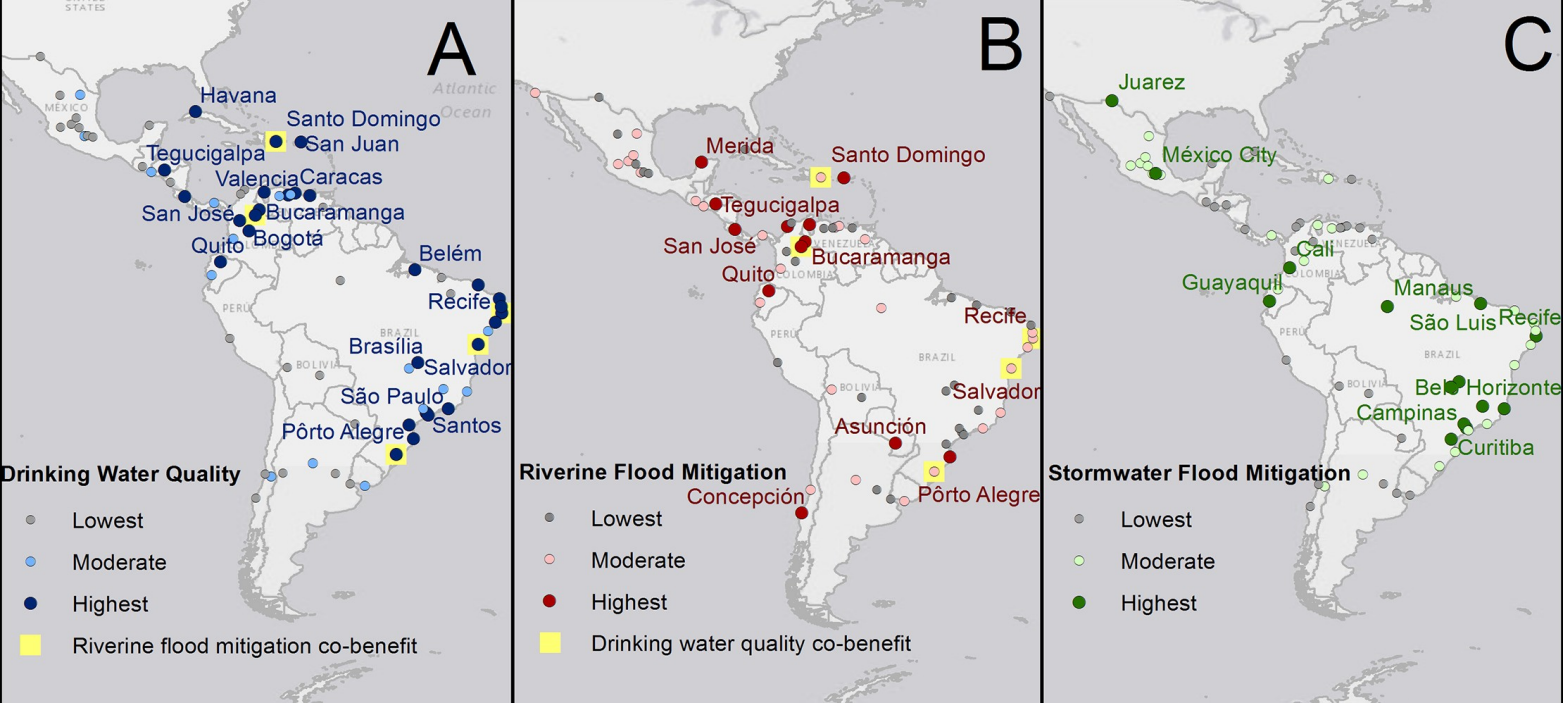
Cities ranked by highest, moderate, and lowest relative opportunity for natural infrastructure. (A) surface drinking water quality improvements, (B) riverine flood risk mitigation, and (C) stormwater flood risk mitigation. City names are shown for the highest opportunity category, and just dots for the moderate and lowest opportunity categories. Cities in which there was overlap of the same servicesheds ranking highest for surface drinking water quality improvements and riverine flood risk mitigation are highlighted in yellow.

If natural infrastructure was used to improve watershed conditions for the highest opportunity cities, this would encompass an estimated population of 96 million people living in these cities, which represents 61% of the total population (around 160 million) across the large cities we analyzed. The potential opportunity varies widely, however, across cities and the three water security dimensions we evaluated (Fig 2). The complete set of metrics and city results for drinking water quality, riverine flooding, and stormwater flooding are available in a data visualization dashboard (http://nascience.us/water-security/), which facilitates a city-to-city relative comparison across the region (e.g., for the Latin America Conservation Council, described earlier, which we engaged as a test case for this analysis).

**Fig 3:**
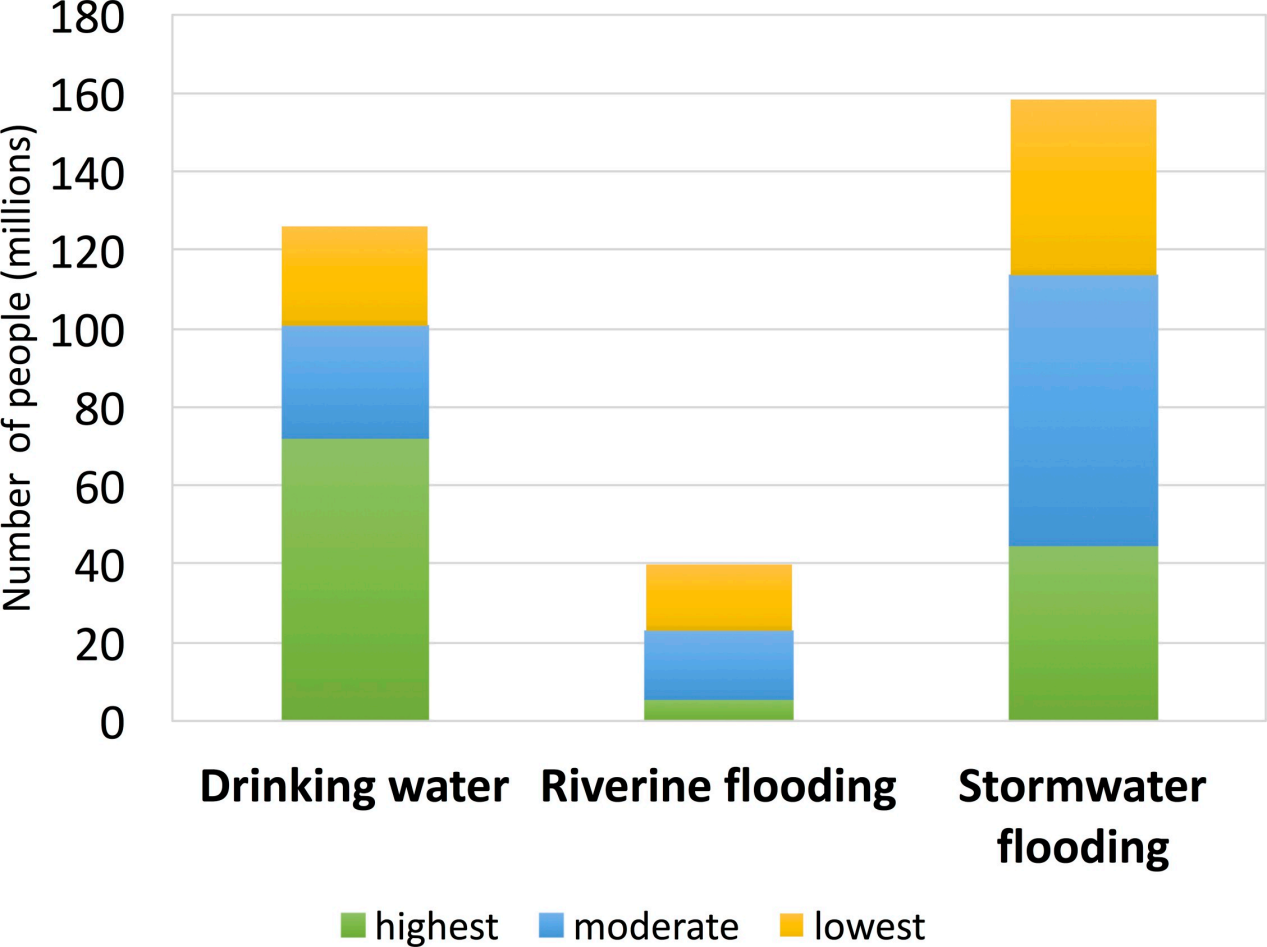
Estimated number of people exposed to surface drinking water quality or flood risk and opportunities for mitigation with natural infrastructure investments.

When considering each of the three analyzed dimensions of water security individually, the number of people living in the highest opportunity cities includes 72 million people across 27 cities who could potentially benefit from water quality improvements, over 5 million people across 13 cities from riverine flood mitigation, and around 44 million people in 14 cities from stormwater flood mitigation (Fig 3). We purposefully use the word ‘potential’, recognizing that our approach is a first-level prioritization screen designed to guide strategy; monitoring and evaluation of actual project implementation is needed over time to determine if potential benefits actually accrue.

Each city has a unique opportunity portfolio based on contrasting biophysical characteristics that generates the potential to reduce one or in some cases two water security risks. Notably, no cities were classified as having the highest opportunity to mitigate all three water security aspects. Only 9 cities, or around 10% of cities in the analysis, had the highest relative opportunity to mitigate two of the three water risks analyzed. These include Bucaramanga, Colombia; Maracaibo, Venezuela; Cucuta, Curitiba, Recife, and São Paolo, Brazil; Quito, Ecuador; Tegucigalpa, Honduras and San Juan., Puerto Rico. No apparent trends were found for this grouping of cities, though for the most part they are mid-range population (between 1–3 million, save São Paolo) and in the humid tropics (save Quito). Interestingly, no cities were ranking in the highest opportunity to mitigate both stormwater and riverine flood water risks.

There was moderate opportunity for surface drinking water quality improvements for an estimated 28 million urbanites (16 cities), moderate river flood exposure mitigation opportunity for 18 million people (28 cities), and moderate stormwater flood mitigation opportunity for 69 million people (27 cities). The lowest opportunity cities for potential drinking water enhancements encompass 25 million people relying on surface water (27 cities), river flood exposure for 25 million people (27 cities), and stormwater flood concerns for 45 million people (27 cities). The populations living in cities in the low opportunity category are unlikely to benefit at the scale from natural infrastructure risk mitigation. Because our ranking is relative, this should not be interpreted as implying that no absolute benefit can be obtained; rather, in a prioritization exercise, relative returns are expected to be lower for cities in this category compared to the higher ranked cities.

While there were 20 cities (around a quarter of all 70 cities analyzed) where floodsheds and source drinking watersheds spatially overlapped by over 50%, there were only five cities for which this overlap occurred in the same servicesheds where natural infrastructure interventions are predicted to have high opportunity to both mitigate flood risk and improve water quality. There are an estimated 14 million people that could potentially benefit from both improved water quality and reduced flood exposure in the cities of Bucaramanga, Colombia; Salvador, Recife, and Porto Alegre, Brazil; and Santo Domingo, Dominican Republic (Fig 2; see cities highlighted in yellow).

For six cities in the analysis, natural infrastructure interventions are modeled to have negligible potential benefits for surface drinking water quality or flood mitigation. Benefits can be negligible to drinking water for cities sourcing mainly from groundwater (e.g., Torreón, Mexico, and Santa Cruz de La Sierra, Bolivia) or for cities where drinking water and flooding originate in very large watersheds that would require large investments to achieve meaningful reductions in pollutant concentration or increases in infiltration (e.g., Barranquilla, Colombia, with the Magdalena River Basin watershed of 260,000 km^2^ or Rosario, Argentina, with the 3.2 million km^2^ Paraná River basin). Other cities like Lima, Peru and Medellín, Colombia have high slopes and low potential for increased infiltration rates due to soil type, which make them poor candidates for flood infrastructure mitigation through natural infrastructure.

## Discussion

The framework developed and applied in this study is science-based yet simple and efficient. It demonstrates the feasibility of producing standardized, empirically-based city-to-city rankings to guide high-level prioritization decisions about watershed natural infrastructure interventions, foremost for those directing efforts at regional and continental scales. The results suggest the substantial scope for potential natural infrastructure investments in Latin America, with at least 96 million people potentially benefiting from enhanced water security related to at least one of the three water security dimensions analyzed. Note that this scope, while significant, is not universal: 61% of Latin American urban dwellers reside in cities where watershed conservation activities is projected to most substantially increase water security based on our analysis. Including additional cities that have moderate opportunity to increase water security, an additional 36 million people could potentially benefit, bringing the overall scope of intervention to nearly 133 million people, or 83% of city dwellers in Latin America’s largest cities.

Our approach is designed as a first-level prioritization for natural infrastructure targeting, but it is not designed to determine if natural infrastructure is sufficient on its own to deliver outcomes desired by cities. In many cases, if not all, cities may require integrating nature-based and conventional engineering approaches to cost-effectively manage water security risks. However, we focus here on ranking natural infrastructure potential at regional scales to fill a critical gap in information that has limited decision makers in the water sector from adopting nature-based approaches to increase water security [18].

**Fig 4:**
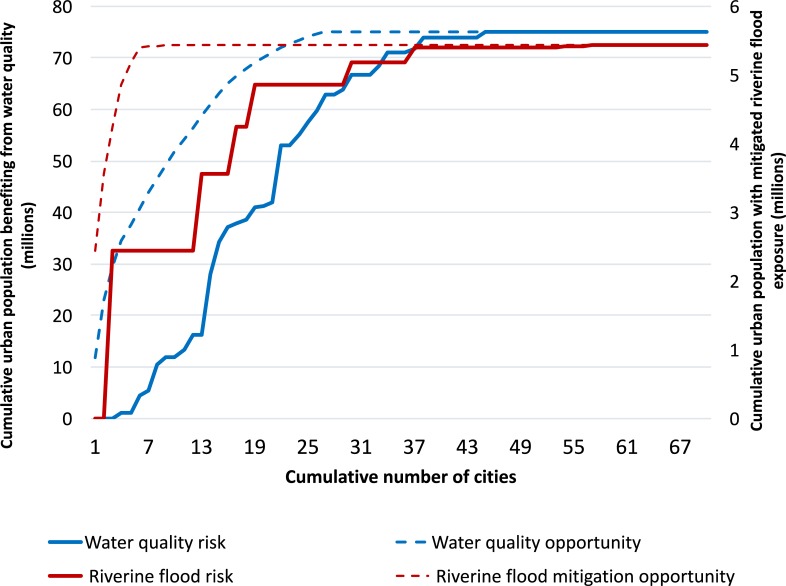
Estimated cumulative number of people potentially benefitting with each additional city based on relative city rankings for risk versus opportunity scores. Population numbers are only counted in cities with the highest opportunity for natural infrastructure based on the regional analysis.

We found that great efficiencies could be achieved by prioritizing cities across the region based on those that have the highest relative opportunity to mitigate risks, instead of selecting cities based solely on their risk level (without considering whether there is strong modeled potential to mitigate that risk). Fig 4 compares the number of cumulative beneficiaries from increased water security for drinking water and riverine flood risk when investing in each additional city based on prioritizing opportunity (dotted line) versus risk (dashed line). Stormwater flooding was not included in Fig 4, because data regarding risk exposure per city is inadequate.

For the purpose of illustration, assume an international development agency has a budget to invest in natural infrastructure to mitigate floods and improve water security in only five cities each (ten cities total), and natural infrastructure carries approximately the same cost per km^2^. If decision makers target the top five cities for natural infrastructure based on flood *risk*, our results indicate only one of these five cities (San Juan, Puerto Rico) would significantly reduce flood risk through natural infrastructure. In this scenario, only 2.4 million people would benefit from natural infrastructure interventions, which is half the number of beneficiaries that could be reached (over 5 million) had decision makers selected cities based on *opportunity* for riverine flood risk mitigation under the same constraints.

Likewise, investing in natural infrastructure to improve drinking water quality for the top five cities with water quality risk would only benefit an estimated 1.2 million people. Yet if a decision maker were to invest in the 5 highest opportunities cities instead, an estimated 30.8 million people would benefit from improved drinking water quality. Over 30 times the number of people could benefit from natural infrastructure if the decision maker targets opportunity instead of risk for the same level of investment.

Continental-scale information on natural infrastructure opportunities could significantly improve the targeting of watershed activities for maximum potential benefit (Fig 4). For instance, decision-makers might consider natural infrastructure investment in cities most at-risk for drinking water or flooding without considering if this approach could have a measurable impact on mitigating these risks. The results suggest that focusing investment based on opportunity metrics constitutes a strategic way to have the greatest marginal impact in terms of number of beneficiaries per targeted city (e.g., including informing the LACC’s strategy to meet its goal of benefitting urban residents across Latin America). Our city ranking for high opportunity cities for improve drinking water quality are consistent with previous analysis, referenced in the introduction which is based on a similar model and data (From the Urban Water Blueprint)[14]. We are unaware of previous work for regional scale analysis of natural infrastructure opportunity to mitigate flood risk, and thus could not compare our results to previous work. Our paper represents a novel analysis for analyzing flood risk and natural infrastructure for a large number of cities.

Cities that have the highest opportunity to mitigate one type of water security risk often do not have highest opportunity to mitigate another (Fig 2). In fact, these “co-benefits” can only be achieved for five cities in the analysis. This is because cities often pipe surface drinking water from smaller, more distant watersheds that do not drain into the urban extent, and thus are not part of a city’s floodshed. Even floodsheds and sewersheds vary, especially for megacities like Mexico City or São Paulo, which do not have enough contiguous non-urbanized area in their respective floodsheds to reduce river flood peaks. Yet, flat slopes and dispersed green space in the sewershed increase the potential that investing in natural infrastructure could reduce stormwater flooding and pressure on the sewer system. Understanding these distinct serviceshed locations and their comparative biophysical conditions is key to evaluating the potential overlap between areas of potential risk mitigation, and whether natural infrastructure activities in one watershed could potentially deliver multiple water security (or other) benefits to a city. For Latin America, our results based upon biophysical characteristics suggest that the best cities to prioritize natural infrastructure activities for improvements to surface drinking water quality are generally not the same cities prioritized for flood mitigation.

One general trend across all three water security dimensions is that, all else equal, natural infrastructure interventions in small source watersheds require a smaller area of conservation action than large watersheds. For surface drinking water quality protection, conservation actions on more hectares are required to change pollutant concentration as watershed size increases. In larger source watersheds, the overall pollutant loading tends to be high, and restoration of expansive areas is required to meaningfully reduce this pollution. Similarly, for riverine and stormwater flooding, conservation actions on larger areas are required to reduce peak discharge as watershed size increases. For natural infrastructure programs designed to mitigate the risks considered in this paper, the most promising context for mitigation investments, represented by 42 cities examined, is a small watershed in which (1) a large number of urban dwellers are exposed to water security risks and (2) partially developed watersheds with substantial areas of agro-pastoral or forest lands, where a relatively small amount of conservation action can potentially improve conditions for a large number of urban residents. We emphasize that our ranking only reveals the relative position of a given city compared to others. Higher ranking cities may be able to substantially improve water security using a greater reliance on natural infrastructure, whereas lower ranking cities may require a primary focus on conventional infrastructure, such as water filtration technology or levee construction to improve urban water security. Many cities in this assessment could benefit from blended natural and conventional infrastructure solutions in their water security portfolio. This is an emerging area where more research is needed [12,15], building on case studies [79,80] and a decision-making framework [81].

Our analytical framework was designed to provide an efficient and systematic assessment of a large number of cities at a continental scale using available regional and global datasets to rank the potential opportunity for natural infrastructure activities based on key biophysical characteristics known to influence effectiveness. This approach was taken to provide decision-relevant information at the geographic scale of interest of national and transnational actors. To maintain simplicity, however, various considerations were omitted owing to data limitations or to inadequate means, at this time, to incorporate them into a systematic comparative prioritization framework. Omissions included built infrastructure affecting the serviceshed, such as dams, reservoirs, enhanced ground water capturing improvements, and water storage infrastructure. Additional omissions include transaction costs of both land and resource owners in the serviceshed and regional and municipal agencies, water utilities, and national laws affecting water and flooding [82,83]. An additional limitation was inadequate continental scale data on best management practices already existing for natural infrastructure flood mitigation, especially rain gardens and efforts to increase infiltration that are not captured by existing land covers. Projects to mitigate flooding could be inflated in watersheds with many best management practices that are not included in this model. All best management practices for flood mitigation are weighted equally absent evidence to suggest otherwise. Future research to link each natural infrastructure strategy to reductions in flood area and exposure is warranted to improve this component.

While our methodology facilitates a first-level prioritization of the potential opportunity for natural infrastructure effectiveness from a biophysical perspective, more research is needed into the institutional characteristics that will facilitate success–and the ability to systematically represent this knowledge in spatial data across a set of cities being analyzed. For instance, institutional structures at municipal agencies or water utilities may limit or enhance the likelihood of natural infrastructure actions being adopted and effective. Similarly, national laws or policy related to clean drinking water standards and development in flood-prone areas will significantly alter the natural infrastructure investment opportunity. Additional factors, such as land tenure, equity, and environmental justice, are also important to consider in urban natural infrastructure [84]. Land tenure in particular must be considered in Latin American cities, where up to one-quarter of the urban area is informal settlement [85]. Building on existing research (e.g., [18,86]) on how institutional factors affect natural infrastructure program formation would allow future regional assessments of natural infrastructure opportunity to include variables that capture these important dimensions. Regardless, however, even if institutional conditions are good for natural infrastructure implementation, if the biophysical characteristics of the serviceshed in question are unconducive, the opportunity to mitigate risk will remain low.

Future research to build upon this analysis would be to analyze the important contributions that natural infrastructure could make to human health, microclimate regulation, recreation, and other ecosystem services that support human well-being in cities. Finally, this analysis represents a current snapshot in time, and additional analyses of how future trends in climate change and urbanization will influence water risk are an important future extension.

## Conclusion

Various research continues to document threats, degradation, and vulnerabilities at multiple scales that are important in identifying people and places at risk to hazards such as poor drinking water quality and flooding [87,88]. Likewise, various research has led to accurate estimations of mitigation opportunities for natural and built infrastructure at local or site-specific scales [89–92]. Despite these achievements, a simple but efficient means to address the role of natural infrastructure that permits comparisons among a large number of locations at regional and continental scales has been heretofore inadequate. Our approach is a novel attempt to fill this methodological and decision-support gap, with a regional demonstration conducted for 70 large cities in Latin America. The results provide LACC and other decision-making units, such as development banks, corporations, and national governments, information to support a high-level prioritization screen for where efforts to target watershed conservation activities could be most effective and potentially benefit the greatest number of people across the region. It is important to emphasize that while our analysis identified many cities where natural infrastructure actions will have negligible or relatively small benefits, or will only mitigate one type of water security risk, final decisions about where to invest should always be vetted against other relevant data and issues not considered in our framework. More inclusive frameworks at continental scales can build from this effort.
